# Co-Occurrence of Cytogenetic Abnormalities and High-Risk Disease in Newly Diagnosed and Relapsed/Refractory Multiple Myeloma

**DOI:** 10.1200/JCO-24-01253

**Published:** 2025-02-18

**Authors:** Martin F. Kaiser, Pieter Sonneveld, David A. Cairns, Marc S. Raab, Jesús San-Miguel Izquierdo, Rick Zhang, Jorge Acosta, Alessandra Larocca, Rakesh Popat, Cong Li, Marc-A. Baertsch, Sarah R. Brown, JuanJose Lahuerta Palacios, Anita K. Gandhi, Sandrine Macé, Pellegrino Musto, Kwee Yong, Elias K. Mai, Franck Dubin, Joan Blade, Andrea Capra, Gordon Cook, Uta Bertsch, María-Victoria Mateos, Mario Boccadoro, Graham H. Jackson, Norma C. Gutiérrez, Francesca Gay, Niels Weinhold

**Affiliations:** ^1^ The Royal Marsden Hospital NHS Foundation Trust, London, United Kingdom; ^2^ Myeloma Molecular Therapy Group, The Institute of Cancer Research, London, United Kingdom; ^3^ Department of Hematology, Erasmus University Medical Center, Rotterdam, the Netherlands; ^4^ Leeds Cancer Research UK Clinical Trials Unit, University of Leeds, Leeds, United Kingdom; ^5^ Internal Medicine V, Hematology, Oncology and Rheumatology, Heidelberg University Hospital, Heidelberg, Germany; ^6^ Clinica Universidad de Navarra, Pamplona, Spain; ^7^ Sanofi, Cambridge, MA; ^8^ Clinical Development Haematology, Celgene International Sàrl, a Bristol Myers Squibb Company, Boudry, Switzerland; ^9^ Division of Hematology, Department of Molecular Biotechnology and Health Sciences, University of Torino, Torino, Italy; ^10^ Department of Haematology, University College London, London, United Kingdom; ^11^ Takeda Development Center Americas, Inc, Deerfield, IL; ^12^ Instituto de Investigación Hospital Universitario 12 de Octubre, Madrid, Spain; ^13^ Hematology Translational Medicine, Bristol Myers Squibb, Summit, NJ; ^14^ Sanofi, Vitry-sur-Seine, France; ^15^ Department of Precision and Regenerative Medicine and Ionian Area, “Aldo Moro” University School of Medicine, Unit of Hematology and Stem Cell Transplantation, AOUC Policlinico, Bari, Italy; ^16^ Hematology Department, Hospital Clinic, Institut d'Investigacions Biomédiques August Pi I Sunyer (IDIBAPS), Barcelona, Spain; ^17^ European Myeloma Network Trial Office, Torino, Italy; ^18^ Leeds Cancer Centre, St James's University Hospital, Leeds, United Kingdom; ^19^ Haematology Department, University of Salamanca, Salamanca, Spain; ^20^ Department of Molecular Biotechnology and Health Sciences, University of Torino, Torino, Italy; ^21^ Department of Haematology, University of Newcastle, Newcastle upon Tyne, United Kingdom; ^22^ Department of Hematology, University Hospital of Salamanca, IBSAL, Cancer Research Center-IBMCC (USAL-CSIC), CIBERONC, Salamanca, Spain; ^23^ Division of Hematology 1, AOU Città della Salute e della Scienza, Department of Molecular Biotechnology and Health Sciences, University of Torino, Torino, Italy

## Abstract

**PURPOSE:**

Survival for patients with multiple myeloma (MM) has improved but outcomes remain heterogeneous. Consistent diagnostic identification of high-risk disease is desirable to address unmet patient need. The aim was to investigate the consistency of association of co-occurrence of high-risk cytogenetic abnormalities (HRCAs) with prognosis in patients with newly diagnosed MM (NDMM) and relapsed/refractory MM (RRMM), and across a range of treatment modalities.

**METHODS:**

A systematic review of randomized controlled trials of MM that reported testing for HRCA between January 1, 2000, and December 9, 2021, was performed. Groups were contacted and asked to locally perform a novel, federated analysis of their data for single hit (one HRCA) and double hit (≥two HRCAs), using a centrally provided algorithm. Analysis results were centrally collated and meta-analyzed to assess the hazard ratio (HR) for progression-free survival (PFS) and overall survival (OS) for one/≥two HRCAs across patient subgroups using random-effects models.

**RESULTS:**

Twenty-four trials including 13,926 patients were included. The median age of participants was 66.5 years (IQR, 59-72) and 56.5% were male (IQR, 52-60). The HR for PFS was 2.28 (95% CI, 2.05 to 2.54) for patients with ≥two HRCAs and 1.51 (95% CI, 1.38 to 1.65) for patients with one HRCA. The HR for OS was 2.94 (95% CI, 2.49 to 3.47) and 1.69 (95% CI, 1.52 to 1.88) for the two subgroups, respectively. In studies initiated since 2015, the effect abides (≥two HRCA PFS, HR, 2.39 [95% CI, 1.96 to 2.91]; OS, 3.10 [95% CI, 2.10 to 4.60]) both for NDMM and RRMM. Heterogeneity related to transplant eligibility and relapsed/refractory status was as expected.

**CONCLUSION:**

The association of ≥two HRCAs with the poorest outcome in NDMM and RRMM, and across treatment modalities, as demonstrated here for the first time to our knowledge, allows for more focused development of novel approaches to these patients with high unmet need.

## INTRODUCTION

Survival of patients with multiple myeloma (MM) has markedly improved over the past 20 years. However, outcomes remain highly heterogeneous with approximately 20% of patients experiencing a rapid relapse within the first 2-3 years after initiation of treatment, also termed high-risk MM (HRMM). The increasing availability of highly active regimens, but also constraints to their access in public health care systems, makes consistent identification of patients with high-risk disease at diagnosis and relapse highly desirable: it holds the prospect of focusing on unmet patient needs within and outside of clinical trials.

**CONTEXT**

**Key Objective**
Consistent diagnostic identification of patients with high-risk multiple myeloma (HRMM) is important for standard-of-care treatment allocation and clinical research.
**Knowledge Generated**
In this federated analysis of clinical trials, ≥two high-risk chromosomal aberrations (HRCAs) were consistently associated with adverse outcome across newly diagnosed, transplant-eligible and transplant-ineligible, and relapsed MM. Strikingly, the prognostic impact of ≥two HRCAs was maintained in trials initiated in the past decade with modern combination therapies, highlighting the ongoing unmet need of HRMM.
**Relevance *(S. Lentzsch)***
This study establishes ≥two HRCAs as a consistent diagnostic marker for HRMM, identifying patients with the poorest outcomes who require intensified treatment strategies. By providing a standardized framework for reporting HRMM in clinical trials, it enables better comparability of therapeutic outcomes and guides the development of targeted interventions for this high-risk group.*
*Relevance section written by *JCO* Associate Editor Suzanne Lentzsch, MD, PhD.

Molecular risk reporting by the International Myeloma Working Group consensus^1^ has so far been limited to the high-risk cytogenetic abnormalities (HRCAs) t(4;14), t(14;16), and del(17p), but recent research suggests genetic profiling including gain(1q) as prognostically relevant, subsequently termed extended genetic profiling. However, association of individual risk markers with outcome is relatively weak and has been inconsistent, suggesting limited certainty in identifying HRMM in trials and clinical care.^2^ Recent evidence in newly diagnosed MM (NDMM) transplant-eligible (TE) patients suggests that co-occurrence of ≥two HRCAs (double hit, or multihit in some contexts) may more consistently identify patients with HRMM, while patients with one HRCA (single hit) may show less adverse and more variable outcome. There is currently a paucity of evidence regarding the prognostic impact of double hit across transplant-ineligible (TNE) and relapsed/refractory MM (RRMM) patients and a wider range of available treatment regimens.

Here, we addressed this uncertainty on the prognostic impact of double hit through identifying randomized, controlled phase II and phase III interventional clinical trials with genetic information, followed by a centrally coordinated federated analysis including in total 13,926 patients with TE and TNE NDMM, and RRMM and subsequent summary-level meta-analysis. In an academic-led initiative, we contacted study groups and industry collaborators who had reported extended genetic profiles from randomized trials to identify studies with at least a complete set of HRCA results including t(4;14), t(14;16), del(17p), and gain(1q) available in a substantial proportion of enrolled patients to ensure representativeness of results. In a federated analysis approach, participating collaborators were requested to perform analyses using uniform, prespecified methods for progression-free survival (PFS) and overall survival (OS) for patient groups defined by the presence of double hit or single hit MM for each trial individually. Federated analysis of individual trials was chosen not only to provide best transparency on potential heterogeneity between data sets, but also to incentivize active participating and closer engagement of all parties with their existing data sets and potential value for systematic genetic stratification of patients.

## METHODS

### Selection Criteria and Search Strategy

For this centrally coordinated and quality controlled federated analysis and meta-analysis, any phase II or III randomized trials reported since 2000 that included PFS and/or OS outcomes and tested for co-occurrence of HRCA were included, for NDMM and RRMM settings. Studies that did not test for HRCA were excluded. The review was not registered.

Studies were identified by searching the bibliographical database PubMed. D.A.C. designed the PubMed search, which sought to identify clinical trials of treatment for MM that reported at least one HRCA to screen for studies that reported multiple HRCAs (Data Supplement, online only). The searches were run on December 9, 2021, and only English language studies were included. A publication date limit of January 1, 2000, to present was applied to all search results. The results of the database searches were downloaded to EndNote (version 20.4.1).

The initial screening (title and abstract) was conducted by one reviewer (D.A.C.). The studies that qualified from first screen underwent a second full-text screen from two reviewers independently (D.A.C. and M.F.K.). Any questions regarding study eligibility were raised to the other reviewer and a consensus reached where possible with the default action being to exclude the study. At this stage, it was identified that data were rarely available in the required format. For studies where the analysis of PFS and OS by no hit (zero HRCA), single hit (one HRCA), and double hit (≥two HRCAs) was not available in the published literature, we contacted the investigators or sponsors. A list of those contacted is given in the Data Supplement (Table S2). The data were recorded in Microsoft Excel (Microsoft Corporation, Redmond, WA) in a worksheet provided by the study statistician (Data Supplement).

### Data Analysis

In a federated analysis approach, participating collaborators provided summaries and performed analyses following uniform, prespecified methods. Academic and industry collaborators provided results generated with these uniform methods. Study design, data specification, analysis and interpretation, and writing of the report were the responsibility of M.F.K., P.S., D.A.C., M.R., F.G., and N.W.

The following descriptive data were requested: trial name, trial registration number, trial patient population (NDMM TE, NDMM TNE, or RRMM), regimens used, patient characteristics in the overall trial population, and in the subset with complete genetic information, including number of patients, median and range of age (years), percentage male sex, and number in International Staging System (ISS) groups^3^ (I, II, III, or not available).

The following genetic data were extracted: the complete information to define no hit, single hit, or double hit including the number of patients with t(4;14), gain(1q) or amp(1q), and del(17p). If t(14;16)/*MAF* translocation testing was performed for all patients with complete genetic information, then this was also included in determining no/single/double hit status. Technical information related to the percentage cutoff for del(17p) positivity, 1q abnormality: how many copies counted as abnormal, and the detection method used was also requested.

Collaborators were requested to locally perform univariate Cox proportional hazard analyses for PFS and OS for patient groups defined by presence of double hit or single hit for each trial individually in a modified intention-to-treat population including those patients with a complete set of genetic results. The following outcome data were extracted for each subgroup defined by single hit or double hit: the estimated hazard ratio (HR) and corresponding 95% CI from Cox proportional hazards models comparing each group with no hit. The number of patients in each trial analysis can be understood by summing the columns no HRCA, one HRCA, and two HRCAs in Table 1. For example, in the model comparing double hit with no hit, you sum no HRCA and two HRCAs. A Wald *P* value from these models for the linear predictor was also extracted. If possible, relevant estimates of median PFS and OS, and estimates at 24 months and 36 months, with corresponding 95% CI estimated using the Kaplan-Meier method were requested. Kaplan-Meier plots for visualization of results were also requested but were described as optional.

**TABLE 1.** Characteristics of the Studies Included in the Meta-Analysis

Study Name	Registration	Years Recruiting	Trial Population	Median Previous Lines	N_ITT_	n_HRCA_	Age, Years, Median (range)	Male (%)	ISS 1 (%)	ISS 2 (%)	ISS 3 (%)	No HRCA	One HRCA	Two HRCAs
MRC-IX	ISRCTN68454111	2003-2007	NDMM (TE)		1,111	511	59 (35-78)	64	23	37	33	266	172	73
HOVON-65/GMMG-HD4	ISRCTN64455289	2005-2008	NDMM (TE)		827	335	57 (25-65)	58	37	32	25	187	106	42
GEM2005MENOS65	ClinicalTrials.gov identifier: NCT00461747	2006-2009	NDMM (TE)		386	218	57 (32-95)	58	38	42	20	123	71	24
GMMG-MM5	ISRCTN05622749	2010-2012	NDMM (TE)		502	524	59 (32-70)	60	38	34	28	259	197	68
NCRI-XI	ClinicalTrials.gov identifier: NCT01554852	2010-2016	NDMM (TE)		2568	1,064	61 (28-75)	62	28	43	23	576	354	134
GEM2012	ClinicalTrials.gov identifier: NCT01916252	2013-2015	NDMM (TE)		458	359	58 (31-65)	52	37	28	29	169	144	46
FORTE	ClinicalTrials.gov identifier: NCT02203643	2015-2017	NDMM (TE)		474	403	57 (32-66)	55	48	35	18	171	144	88
GMMG-HD6	ClinicalTrials.gov identifier: NCT02495922	2015-2017	NDMM (TE)		559	459	59 (25-65)	60	42	37	21	228	153	78
Cardamon	ClinicalTrials.gov identifier: NCT02315716	2015-2019	NDMM (TE)		281	233	59 (34-74)	59	46	37	18	137	73	23
MRC-IX	ISRCTN68454111	2003-2007	NDMM (TNE)		849	358	73 (61-89)	57	11	36	43	181	120	57
GIMEMA-03-05	ClinicalTrials.gov identifier: NCT01063179	2006-2008	NDMM (TNE)		511	130	72 (57-87)	54	23	35	24	53	55	22
GEM2005MAS65	ClinicalTrials.gov identifier: NCT00443235	2006-2008	NDMM (TNE)		260	155	72 (65-84)	47	29	40	42	80	61	14
NCRI-XI	ClinicalTrials.gov identifier: NCT01554852	2010-2016	NDMM (TNE)		1,852	750	74 (56-89)	58	15	41	37	409	273	68
GEM2010MAS65	ClinicalTrials.gov identifier: NCT01237249	2011-2013	NDMM (TNE)		233	150	73 (65-88)	51	28	47	24	70	64	16
RV-MM-PI-0752	ClinicalTrials.gov identifier: NCT02215980	2014-2016	NDMM (TNE)		199	134	76 (67-80)	51	31	44	24	64	49	21
EMN10	ClinicalTrials.gov identifier: NCT02586038	2015-2018	NDMM (TNE)		175	139	74 (53-88)	48	27	46	27	64	56	19
GMMG-ReLApsE	ISRCTN16345835	2010-2016	RRMM	1	277	182	61 (29-74)	59	59	25	10	64	88	30
TOURMALINE-MM1	ClinicalTrials.gov identifier: NCT01564537	2012-2014	RRMM	1	722	529	66 (30-91)	56	63	24	13	236	219	74
MUKFive	ISRCTN17354232	2013-2016	RRMM	1	300	171	68 (40-82)	64	48	37	15	85	71	15
OPTIMISMM	ClinicalTrials.gov identifier: NCT01734928	2013-2017	RRMM	1+	559	350	65 (25-85)	55	51	31	6	166	130	54
MUKSeven	ISRCTN24593488	2016-2018	RRMM	3	102	71	72 (44-85)	65	32	31	37	29	33	9
MUKEight	ISRCTN58227268	2016-2018	RRMM	4	112	48	71 (50-80)	48	21	44	33	20	15	13
ICARIA	ClinicalTrials.gov identifier: NCT02990338	2017-2018	RRMM	2+	307	194	67 (36-86)	52	25	32	28	64	99	31
IKEMA	ClinicalTrials.gov identifier: NCT03275285	2017-2019	RRMM	2+	302	257	64 (33-90)	56	53	31	15	108	105	44

Abbreviations: EMN, European Myeloma Network; GEM, Grupo Español de Mieloma; GIMEMA, Gruppo Italiano Malattie Ematologiche Maligne dell'Adulto; GMMG, German Multicenter Myeloma Group; HOVON, Dutch-Belgian Hemato-Oncology Cooperative Group; HRCA, high-risk cytogenetic abnormality; ISS, International Staging System as assessed at trial entry; MM, multiple myeloma; MRC, Medical Research Council; MUK, Myeloma UK; NCRI, National Cancer Research Institute; NDMM, newly diagnosed MM; N_HRCA_, number with complete data for HRCA; N_ITT_, number in the intention-to-treat population; RRMM, relapsed/refractory MM; TE, transplant-eligible; TNE, transplant-ineligible.

We collated results centrally and performed a meta-analysis using the HR of progression or death for PFS, or death for OS. As the trial populations treated were expected to be heterogeneous, we decided a priori to use a random-effects model using the Der Simonian and Laird method.^4^ Results were presented with forest plots. All analyses were performed using R (version 4.2.1).^5^

We also planned a priori to perform subgroup analysis, considering patients with TE NDMM, TNE NDMM, and RRMM in separate meta-analysis. We undertook sensitivity analysis focusing on studies commencing recruitment since 2015. The funnel plot and the Egger test were used to assess publication and availability bias.^6^ Heterogeneity was assessed with the *I*^2^ statistic^7^ ([*Q* – *df*]/*Q* × 100, where *Q* is the chi-square test for statistical heterogeneity, and *df* its associated degrees of freedom) and 50% or more was defined as substantial heterogeneity on the basis of the Cochrane Handbook for Systematic Reviews of Interventions.^8^

### Role of the Funding Source

The funders of the study had no role in study design, data collection, data analysis, data interpretation, or writing of the report. Industry partners were invited collaborators, provided no funding, and had no role in study design or data interpretation.

## RESULTS

### Search and Data Extraction

**FIG 1.**
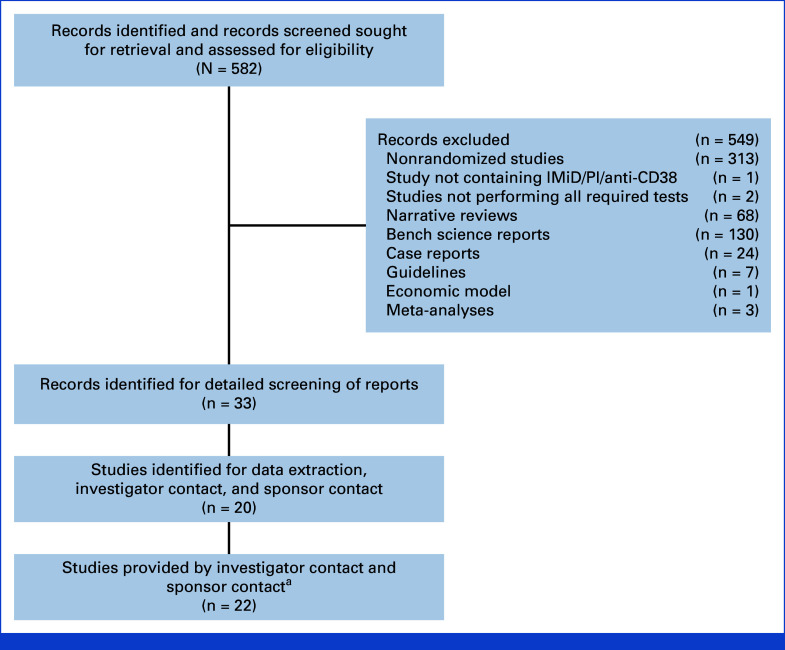
Study selection. ^a^Two studies (MRC Myeloma IX and NCRI Myeloma XI were platform trials including pathways for transplant-eligible and transplant-ineligible NDMM). IMiD, immunomodulatory drug; MRC, Medical Research Council; NCRI, National Cancer Research Institute; NDMM, newly diagnosed multiple myeloma; PI, proteasome inhibitor.

The search identified 582 reports for screening (Fig 1). After screening, 33 reports were included^9-41^ that reported 20 randomized studies identified for further consideration (Data Supplement, Table S1). After contacting investigators and sponsors, nine of these studies were excluded (two sponsors declined to participate totaling eight studies, and one study did not have complete genetic information). However, contact with investigators and sponsors identified 15 further studies that were eligible (Data Supplement, Table S3). Hence, 24 studies comprising 13,926 patients were included.^42-66^ Two studies were included that contained pathways for TE and TNE NDMM: Medical Research Council-IX and National Cancer Research Institute (NCRI) XI. The study characteristics are provided in Table 1 and brief descriptions of the regimens investigated are provided in the Data Supplement (Table S4).

### Study Characteristics

In total, results from the federated analysis on HRCA co-occurrence were successfully provided on 7,724 patients (out of 13,926, 55.5%) enrolled in 24 trials and results as per prespecified patient subgroups were included in the meta-analysis: 4,106 patients from nine trials conducted in TE NDMM, 1,816 from seven trials conducted in TNE NDMM, and 1,802 from eight trials conducted in RRMM. The median age of participants was 66.5 years (IQR, 59-72) and 56.5% were male (IQR, 52-60). The percentage of patients with ISS-I, ISS-II, and ISS-III were 34.5% (IQR, 27-47), 37% (IQR, 34-41), and 24% (IQR, 18-30), respectively. Frequencies of double hit (median, 13.8%; IQR, 12.2-16.1) and single hit (median, 37.4%; IQR, 33.5-41.4) were comparable across trials.

### Meta-Analysis

**FIG 2.**
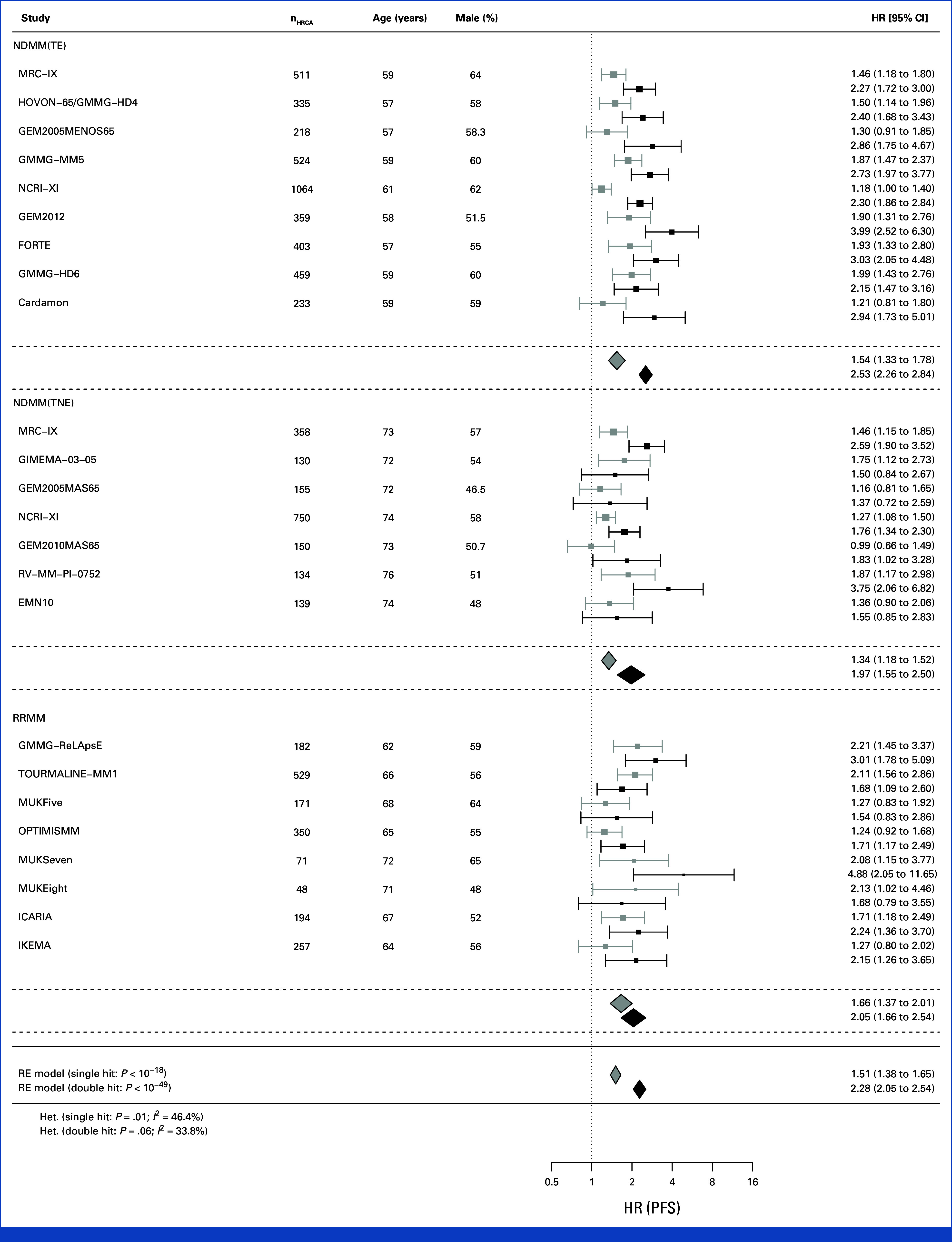
Random-effects meta-analysis of all studies considering PFS, by patient group and overall. EMN, European Myeloma Network; GEM, Grupo Español de Mieloma; GIMEMA, Gruppo Italiano Malattie Ematologiche Maligne dell'Adulto; GMMG, German Multicenter Myeloma Group; HOVON, Dutch-Belgian Hemato-Oncology Cooperative Group; HR, hazard ratio; MRC, Medical Research Council; MM, multiple myeloma; MUK, Myeloma UK; NCRI, National Cancer Research Institute; NDMM, newly diagnosed MM; PFS, progression-free survival; RE, random-effects; RRMM, relapsed/refractory MM; TE, transplant-eligible; TNE, transplant-ineligible.

**FIG 3.**
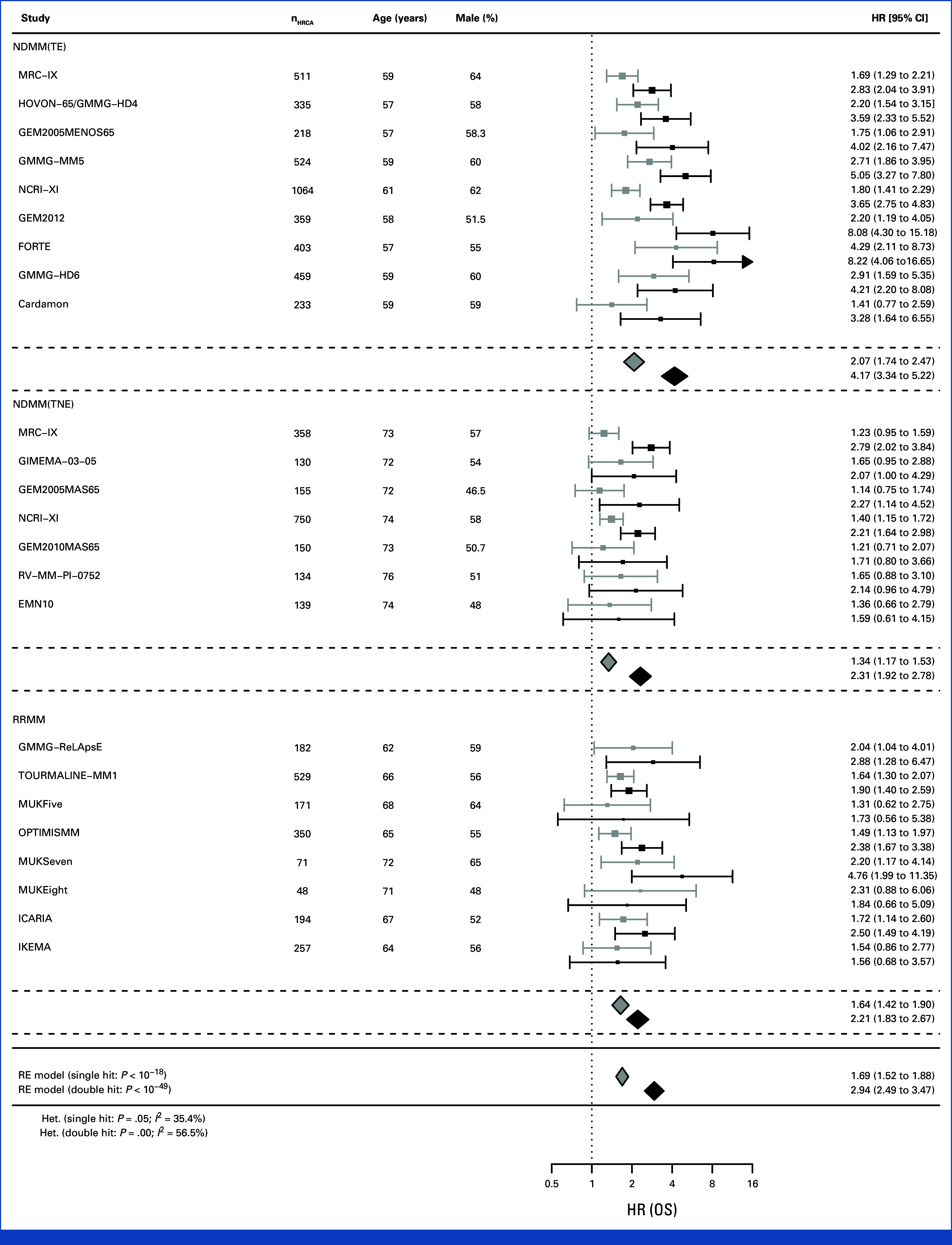
Random-effects meta-analysis of all studies considering OS, by patient group and overall. EMN, European Myeloma Network; GEM, Grupo Español de Mieloma; GIMEMA, Gruppo Italiano Malattie Ematologiche Maligne dell'Adulto; GMMG, German Multicenter Myeloma Group; HOVON, Dutch-Belgian Hemato-Oncology Cooperative Group; HR, hazard ratio; MM, multiple myeloma; MRC, Medical Research Council; MUK, Myeloma UK; NCRI, National Cancer Research Institute; NDMM, newly diagnosed MM; OS, overall survival; RE, random-effects; RRMM, relapsed/refractory MM; TE, transplant-eligible; TNE, transplant-ineligible.

Meta-analysis of all studies showed highly consistent separation of risk groups by co-occurrence of HRCAs: PFS HR for double hit was 2.28 (95% CI, 2.05 to 2.54; *P* < 10^–49^) and for single hit was 1.51 (95% CI, 1.38 to 1.65; *P <* 10^–18^) compared with those without HRCA (Fig 2). The *I*^2^ for PFS for single hit was 46.4% and for double hit was 33.8%. OS HRs were 2.94 (95% CI, 2.49 to 3.47; *P* < 10^–36^) and 1.69 (95% CI, 1.52 to 1.86; *P* < 10^–21^), respectively, compared with those without HRCA (Fig 3). The *I*^2^ for OS was 35.4% for single hit and 56.5% for double hit, which is substantial by accepted definitions.^7^ However, 95% CIs did not overlap between double hit and single hit groups, providing evidence for consistent prognostic discrimination by number of HRCAs.

Considering clinical subgroups separately, double hit MM was consistently associated with the most adverse outcomes across patients with TE NDMM, TNE NDMM, and RRMM. The prognostic effect size was largest in patients with TE NDMM (PFS, 2.53 [95% CI, 2.26 to 2.84]; OS HR, 4.17 [95% CI, 3.34 to 5.22]), followed by patients with TNE NDMM (PFS, 1.97 [95% CI, 1.55 to 2.50]; OS HR, 2.31 [95% CI, 1.92 to 2.78]) and patients with RRMM (PFS, 2.05 [95% CI, 1.66 to 2.54]; OS HR, 2.21 [95% CI, 1.83 to 2.67]). In TE NDMM, the *I*^2^ for PFS for single hit was 57.7% and double hit was 0.0%, and for OS for single hit was 36.1% and double hit was 47.3%. In TNE NDMM, the *I*^2^ for PFS for single hit was 14.1% and double hit was 44.8%%, and for OS for single hit was 0.0% and double hit was 0.0%. In RRMM, the *I*^2^ for PFS for single hit was 44.8% and double hit was 39.5%, and for OS for single hit was 0.0% and double hit was 0.0%.

Considering only those studies commencing recruitment since 2015, including 2,312 patients (Data Supplement, Table S4), double hit MM was consistently associated with the most adverse outcomes overall (PFS, 2.39 [95% CI, 1.96 to 2.91]; OS HR, 3.10 [95% CI, 2.10 to 4.60]) and across patients with NDMM (PFS, 2.62 [95% CI, 2.05 to 3.35]; OS HR, 4.81 [95% CI, 2.85 to 8.13]) and RRMM (PFS, 2.34 [95% CI, 1.66 to 3.30]; OS HR, 2.45 [95% CI, 1.61 to 3.73]).

**FIG 4.**
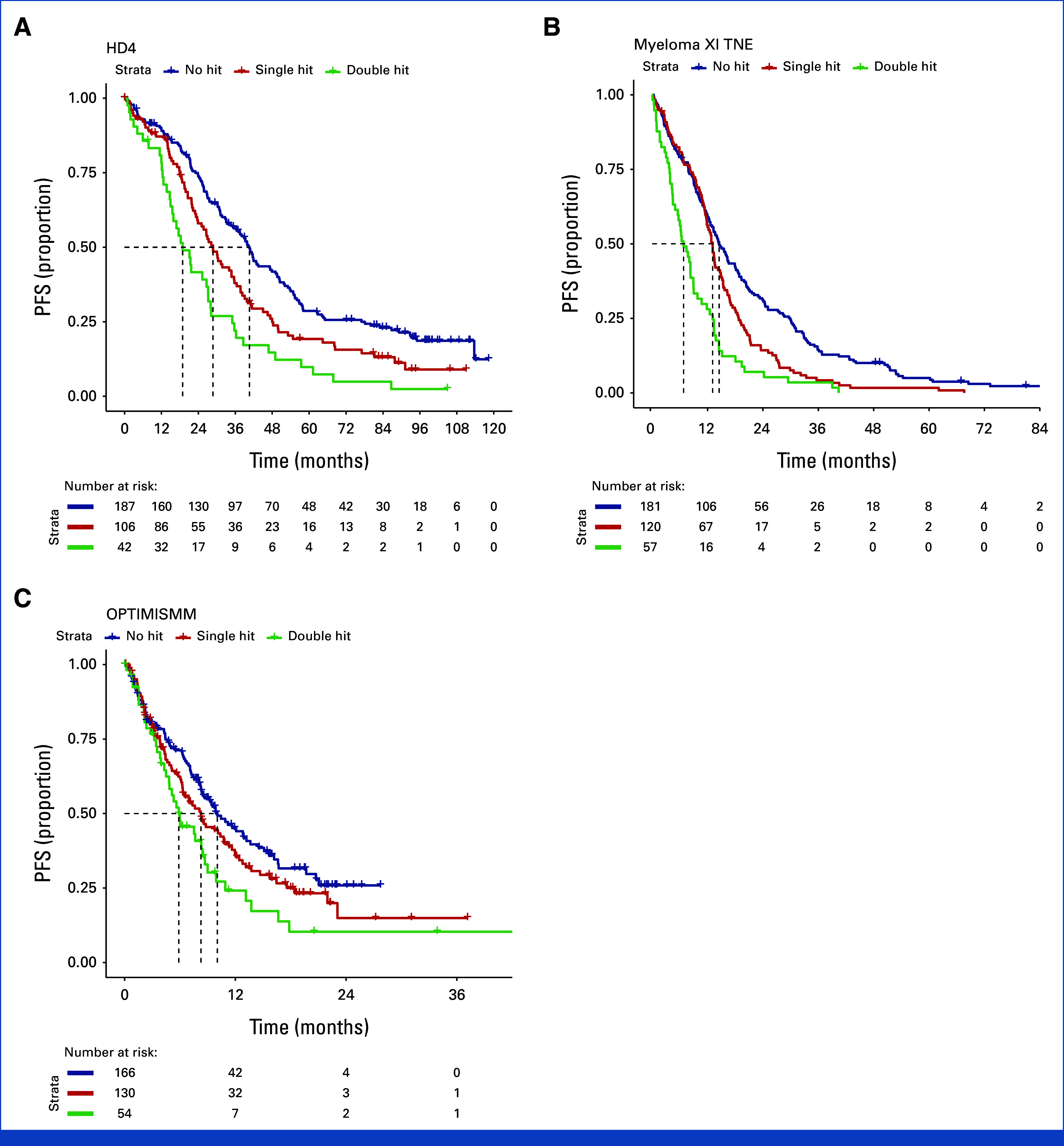
Kaplan-Meier estimates of survivor function for PFS separated by co-occurrence of cytogenetic abnormality for representative trials in (A) NDMM TE—HOVON-65/GMMG-HD4, (B) NDMM TNE—NCRI Myeloma XI, and (C) RRMM—OPTIMISMM. GMMG, German Multicenter Myeloma Group; HOVON, Dutch-Belgian Hemato-Oncology Cooperative Group; MM, multiple myeloma; NCRI, National Cancer Research Institute; NDMM, newly diagnosed MM; PFS, progression-free survival; RRMM, relapsed/refractory MM; TE, transplant-eligible; TNE, transplant-ineligible.

**FIG 5.**
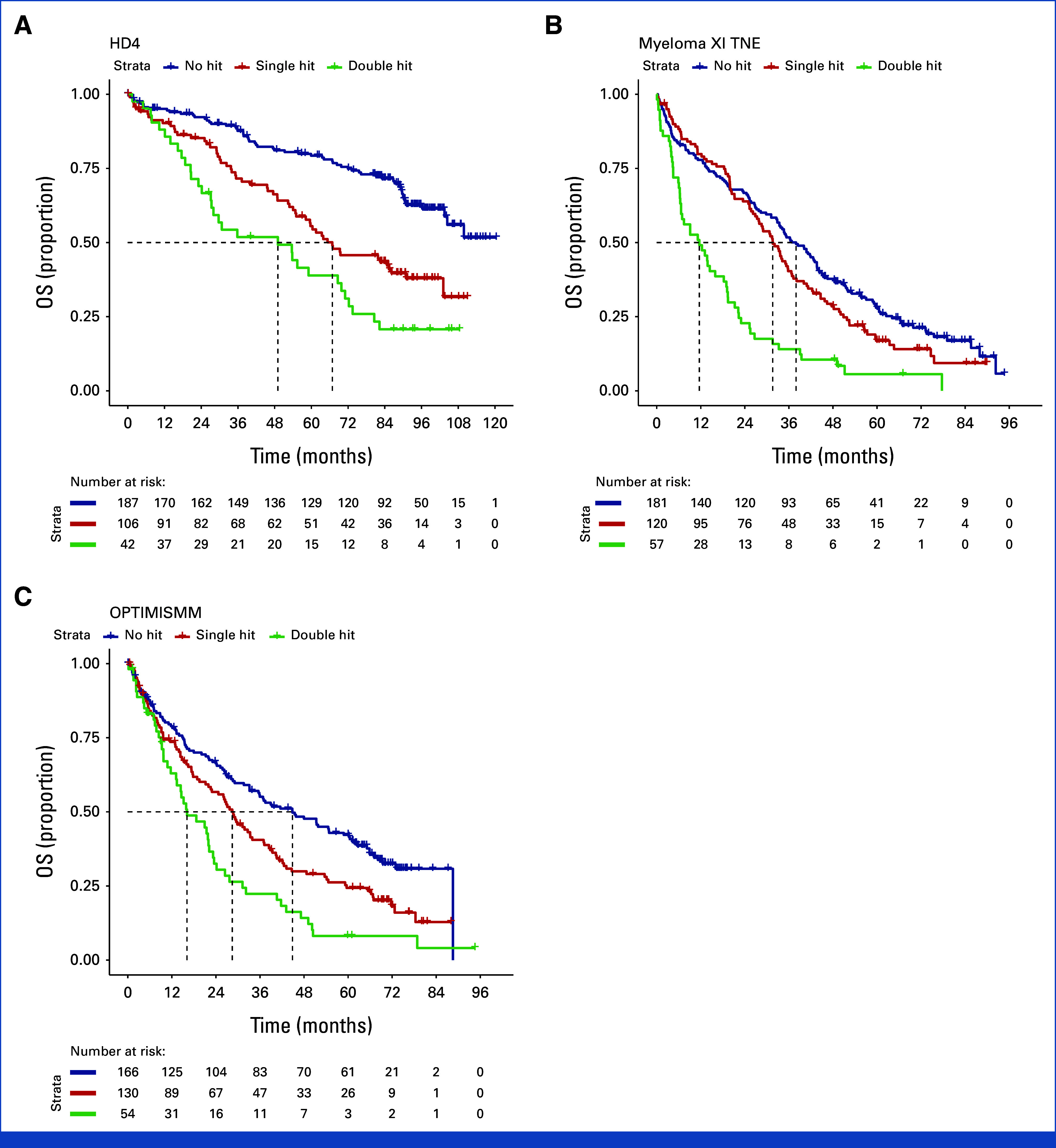
Kaplan-Meier estimates of survivor function for OS separated by co-occurrence of cytogenetic abnormality for representative trials in (A) TE—HOVON-65/GMMG-HD4, (B) TNE—NCRI Myeloma XI, and (C) RRMM—OPTIMISMM. GMMG, German Multicenter Myeloma Group; HOVON, Dutch-Belgian Hemato-Oncology Cooperative Group; NCRI, National Cancer Research Institute; OS, overall survival; TE, transplant-eligible; TNE, transplant-ineligible; RRMM, relapsed/refractory multiple myeloma.

Considering each study separately showed consistent separation of Kaplan-Meier estimated survivor functions by co-occurrence of HRCAs for PFS and OS in double hit and single hit for TE NDMM (Figs 4A and 5A; Data Supplement, Fig S1), TNE NDMM (Figs 4B and 5B; Data Supplement, Fig S2), and RRMM (Figs 4C and 5C; Data Supplement, Fig S3). Of note, the separation was consistently observed in studies considering combination of proteasome inhibitors and immunomodulatory agents such as FORTE for NDMM and OPTIMISMM in RRMM, as well as anti-CD38 monoclonal antibody combination therapy such as in ICARIA.

### Risk of Bias

In terms of publication and availability bias, the funnel plots for PFS (Data Supplement, Fig S4) did not indicate bias, which was confirmed by Egger's test for single hit (*z* = 1.5718, *P* = .1160) and double hit (*z* = –0.0240, *P* = .9809). Findings were similar for TE NDMM (single hit: *z* = 1.0828, *P* = .2789; double hit: 1.8265, *P* = .0678), TNE NDMM (single hit: *z* = 0.5595, *P* = .5758; double hit: *z* = –0.4292, *P* = .6678), and RRMM (single hit: *z* = 0.6195, *P* = .5356; double hit: *z* = 1.4207, *P* = .1554). There was no evidence of asymmetry in the funnel plot that might indicate small-study effect, but NCRI-XI was an example of a large study that provided a smaller effect size than might be expected, particularly in the TE pathway patients. The funnel plots for OS (Data Supplement, Fig S5) did not indicate bias, which was confirmed by Egger's test for single hit (*z* = 1.5988, *P* = .1099) and double hit (*z* = –0.3667, *P* = .7139). Findings were similar for TE NDMM (single hit: *z* = 1.4946, *P* = .1350), TNE NDMM (single hit: *z* = 0.1835, *P* = .8544; double hit: *z* = –1.0793, *P* = .2805), and RRMM (single hit: *z* = 0.7654, *P* = .4440; double hit: *z* = 0.5380, *P* = .5906). However, for TE NDMM double hit, there was evidence of asymmetry supported by Egger's test (double hit: *z* = 1.9754, *P* = .0482). This might indicate smaller-study effects, particularly notable in GEM2012 and FORTE.

## DISCUSSION

Over the past 20 years, evidence has accumulated that certain cytogenetic abnormalities, including del(17p), translocation t(4;14), translocation t(14;16), and gain(1q), are associated with poor prognosis in patients with MM. However, despite the considerable data available from trials and real-world experience, the association between outcome and co-occurrence of cytogenetic abnormalities in different patient groups remains unclear. Our analysis, which included 24 randomized controlled trials comprising 13,926 patients, addresses this question. We show that co-occurrence of two or more cytogenetic abnormalities confers significant poor prognosis in patients with NDMM that were TE or TNE, and in patients with RRMM.

Prognostic association was consistent across trials investigating immunomodulatory, proteasome inhibitor, or anti-CD38 monoclonal antibody therapies and combinations. Effect sizes were consistent in trials commencing since 2015, but among the highest for the most contemporaneous trials such as FORTE (OS HR, 4.29 [95% CI, 2.11 to 8.73]) for TE NDMM, and OPTIMISMM (OS HR, 2.71 [95% CI, 1.76 to 4.17]) and ICARIA (OS HR, 2.50 [95% CI, 1.49 to 4.19]) for RRMM. This may suggest a proportionately higher benefit from current regimens for patients with standard risk and highlights the ongoing unmet need for patients with more aggressive disease, despite the marked improvement of therapies. We observed mildly higher heterogeneity in RRMM trials, which inherently include clinically diverse patient populations; the relevance of co-occurrence of cytogenetic lesions should accordingly be contextualized in these trials with other important prognostic factors, including, but not limited to, exposure and refractoriness to previous treatments. Relatively lower effect sizes in TNE NDMM would be consistent with the previously described higher frequency and impact of frailty and comorbidity on outcome in these patients,^35^ although our study was not designed to investigate these further.

Strengths of our study include the diversity of trials, treatments, and patient populations included in the meta-analysis. We intentionally did not contrast individual treatment arms of the randomized trials, which could incentivize scientifically inappropriate cross-trial comparisons, but designed the study to generate evidence on the usefulness of extended genetic profiling and the co-occurrence of HRCAs. Participating groups rated the novel, federated analysis approach highly, which allowed all teams to gain local expertise and novel insight into their own data, while still contributing to a collaborative aim. A limitation of our study is the absence of trials that include frontline use of anti-CD38 in TE NDMM in the context of triplet and quadruplet combinations. It would also be of interest to include studies that have compared transplant and nontransplant strategies in more depth. However, it is likely that double hit retains its negative prognostic association with PFS and OS, while the impact on single hit is not clear, as demonstrated recently in studies ineligible (nonrandomized)^67^ or unavailable to this meta-analysis (sponsor declined participation).^68^

Our analysis did not set out to incorporate nongenetic prognostic factors such as beta-2-microglobulin, or next-generation sequencing genomic data, nor did it aim to use such information to build a more complex classifier, such as those recently proposed.^69-71^ The analysis did not aim to exclusively and definitively define high risk on co-occurrence of markers only; single genetic lesions can have marked prognostic impact, including those resulting in homozygous inactivation of tumor suppressor genes such as TP53.^72,73^ However, these events are relatively rare, and they have not yet been consistently tested and reported across international NDMM and RRMM trials. We did not consider the type of HRCA, but rather any two or more HRCA. It is possible that additional HRCAs such as del(1p) should be considered as well, and that some combinations of specific HRCAs are worse than others. Furthermore, it would be interesting to understand the impact of lesions acquired in the NDMM to RRMM transition as we explored in a longitudinal study and found a clear added prognostic impact of lesions acquired or detectable for the first time at relapse.^37^ Studies investigating this in more detail are eagerly awaited.

The analysis did aim to evaluate the enhanced utility of combining a set of established, accessible genetic markers across current treatment modalities and indications. We believe that our results strongly support wider patient access to these tests, both within and outside of clinical trials. These results accordingly also form a basis and framework for future detailed and delineated exploration of single hit disease.

The heterogeneity observed in this study reflects the diversity of trial participants and is therefore a strength of our findings. The *I*^2^ statistic is a measure of the impact of heterogeneity on the summary effect estimate. In each of the subgroup meta-analyses undertaken in this report, *I*^2^ is greater than zero and, in the TE NDMM double hit analysis, it can be classified as substantial. However, when considering the clinical subgroups separately, *I*^2^ for PFS and OS for each of single hit and double hit was <50%. This indicates that the observed heterogeneity is due to the diversity of patients in each of these subgroups and the estimates of prognostic association should be considered alongside the estimates of heterogeneity. As a corollary to this, the estimates in each of the patient subgroups are not affected as strongly by heterogeneity, and hence can be considered as such.

In conclusion, co-occurrence of cytogenetic abnormalities is associated with poor prognosis in patients with MM. This association is consistent in key patient subgroups and across widely accessible treatment modalities, across TE NDMM and RRMM, supporting improved access to testing and reporting of risk marker co-occurrence across patient groups.

## Data Availability

All data included in this manuscript are available in tables, figures, and the Data Supplement. Code used to undertake this analysis is available from the authors upon request.
